# Supplementary material for: A subset of viruses thrives following microbial resuscitation during rewetting of a seasonally dry California grassland soil
Source: Nat Commun. 2023 Sep 20;14:5835. doi: 10.1038/s41467-023-40835-4 (PMC10511743; doi:10.1038/s41467-023-40835-4)
Supplement: Supplementary file 1 — Supplementary information [file 41467_2023_40835_MOESM1_ESM.pdf]

SUPPLEMENTARY FIGURES

Supplementary Table 1 | Mixed 'omic methods enable detection and distinguishing of genomes as abundant, active, and virions.

**Metagenome type**	**Method**	**Presumed composition**	**Quantity**
Unfractionated metagenome	Direct DNA extraction from soil (Figure 1A)	Bacterial, archaeal, eukaryotic, and viral sequences from MAGs and vOTUs (which may be intracellular or extracellularized).	24
SIP-fractionated metagenome	DNA extracted from soil and ultracentrifuged in a CsCl gradient to capture distinct DNA-density fractions for quantitative stable isotope probing (qSIP) (Figure 1B)	$H_2^{18}O$-incorporating MAG and vOTU genomes in denser DNA fractions i.e., considered active.	210
Virome	Small-fraction metagenome, i.e. viral-enriched sequences: soil buffered, 0.2 µm filtered, concentrated, treated with DNase. DNA extracted from 0.2 µm effluent (Figure 1A)	Sequences from < 0.2 µm membrane-bound or encapsulated genomes i.e. Viral-like particles (VLPs) or virions; and ultrasmall cells such as CPR bacteria and nanoarchaea	18

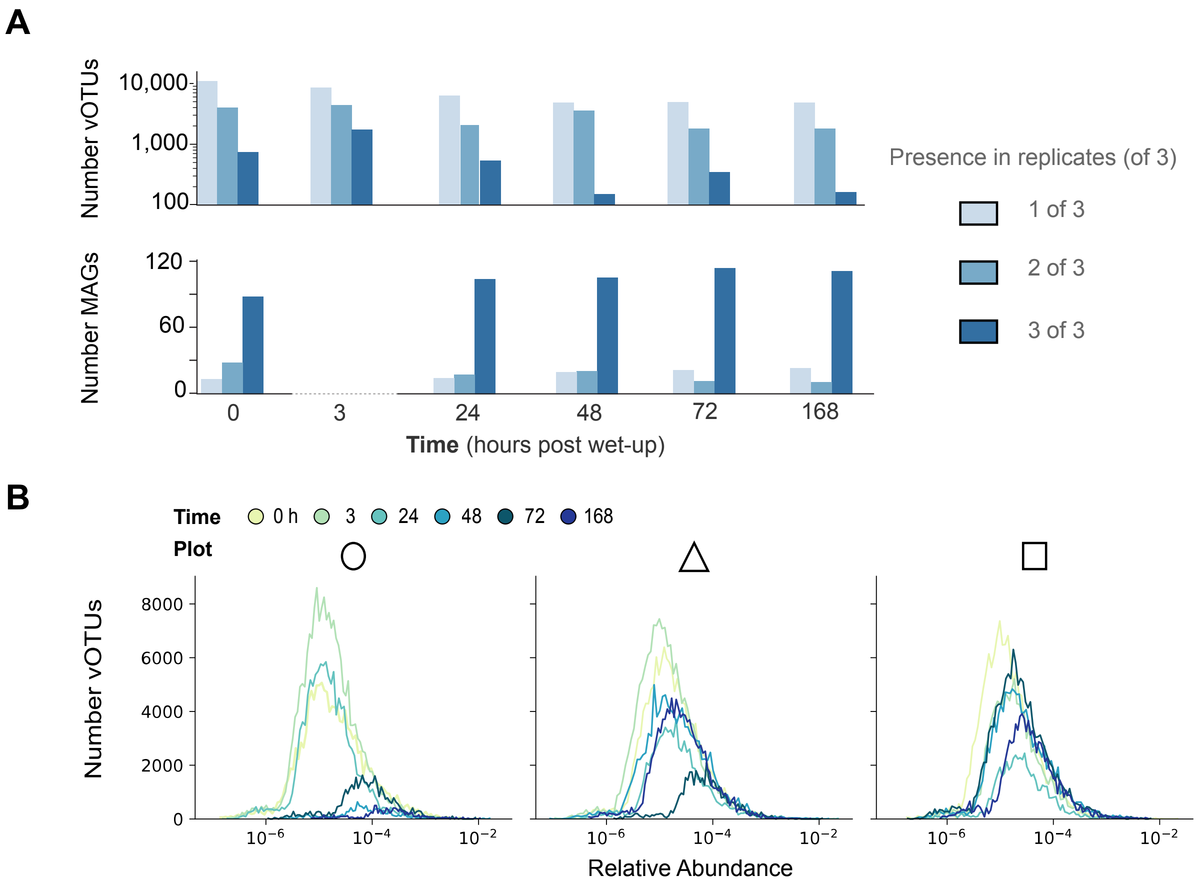

Supplementary Figure 1 | Viral heterogeneity through space. (A) Barplots visualize the number of (top) recovered vOTUs (log scale) or (bottom) MAGS found in one (lightest blue), two, or three (darkest blue) of three replicate microcosms. Metagenomes were collected at 0, 24, 48, 72, 168 hours: dotted line at 3 hours indicates metagenomes were not collected at this time point. (B) Distribution of vOTU relative abundances (log scale) per time point (colored curve) graphed by field plot (signified by circle, triangle, and square used throughout). The y axis represents the number of unique vOTUs detected per time point.

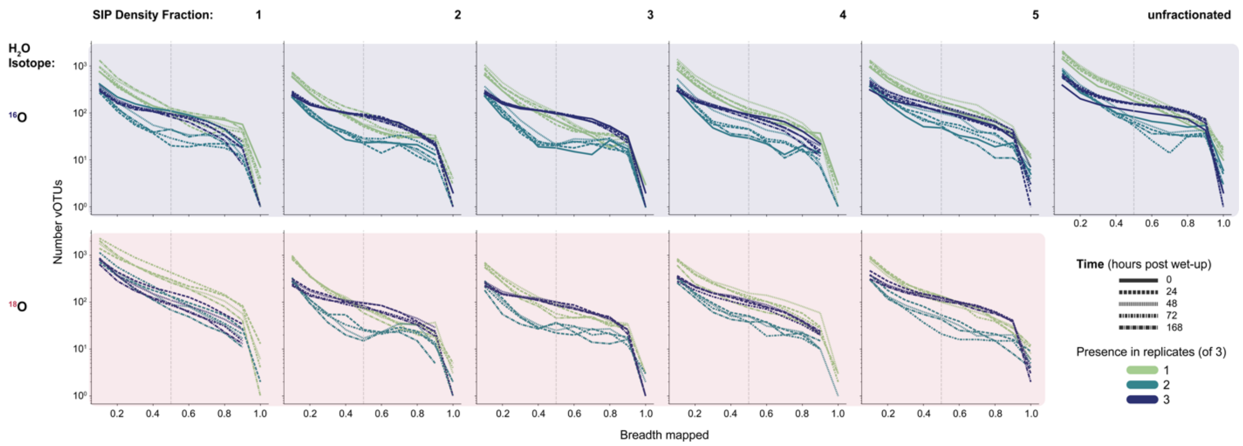

Supplementary Figure 2 | Establishing a breadth cutoff for qSIP. Each plot shows the count of vOTUs (y axis) detected using increasing breadth cutoffs (x axis) per each of five density fractions in the ^{16}O- (blue shaded) compared to ^{18}O-H_2O (red shaded) watering treatments. Only for natural abundant water ($H_2{}^{16}O$) was unfractionated DNA sequenced. The style of line represents timepoints 0, 24, 48, 72, or 168 hours post wet-up. The three distinct colors correspond to the number of microcosms a vOTU appeared in with green signifying the count of vOTUs appearing only once, light blue as the count of vOTUs detected in two of three replicates, and dark blue as vOTUs found in all three replicates. Breadth mapped refers to the coverage of reads recruited to vOTUs to discern whether or not a vOTU is considered detected.

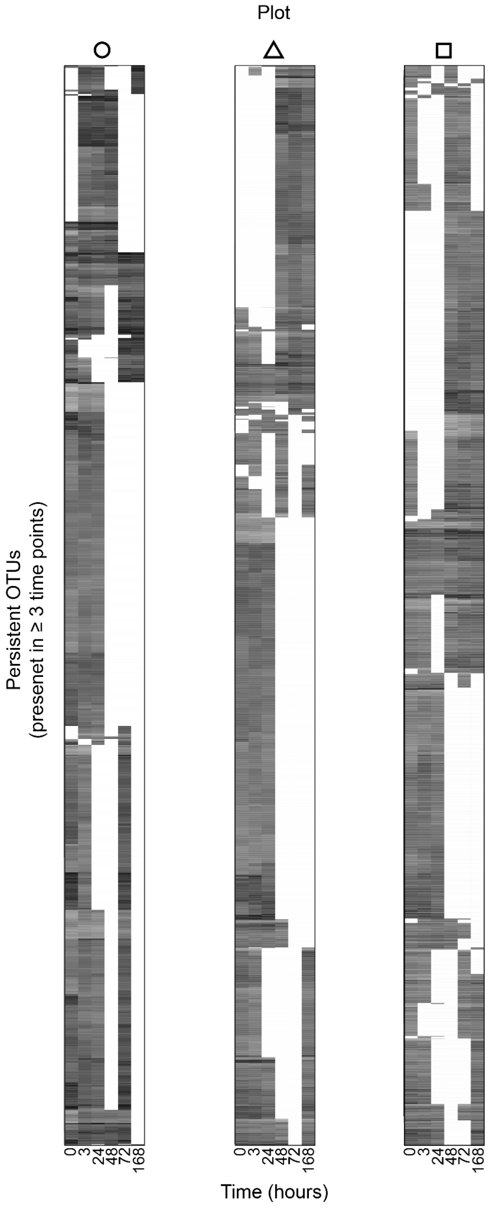

Supplementary Figure 3 | Temporal changes in relative abundance of persistent viral populations (vOTUs). Each heatmap corresponds to one of the three field plots sampled and is hierarchically clustered according to vOTU relative abundance (rows are not shared between heatmaps). Time is shown on the x-axis of each plot (columns); Each row (y-axis) corresponds to a unique vOTU and its relative abundance through time. All vOTUs were present in each given plot in at least three time points, i.e., “persistent” vOTUs. Circles, triangles, and squares correspond to the field plots in the PCoA legend (Figure 2).

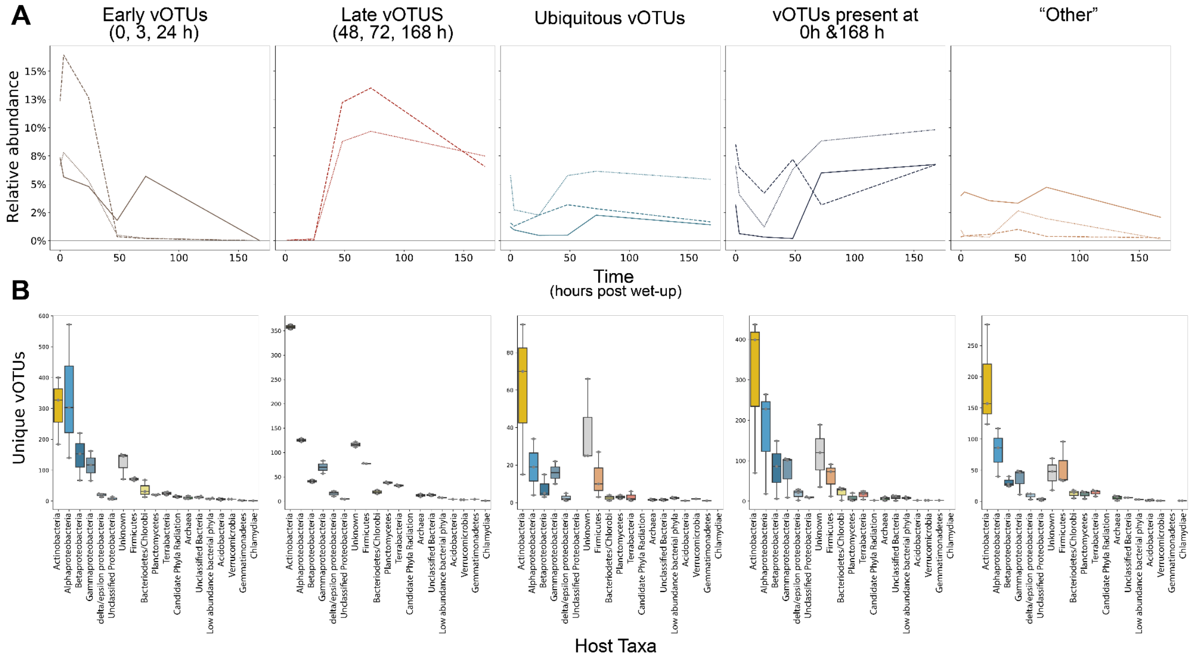

Supplementary Figure 4 | Viral dynamics following soil wet-up classified by temporal response. (A) Each plot shows the aggregated relative abundance per plot of the persistent vOTUs that respond early (0, 3, 24h); late (48, 72, 168h); ubiquitously (at all time points); present at 0h, 168h, and one other time point; and other. Relative abundance is shown as the percent of total reads per virome. Colors correspond to viral temporal response category. **(B)** Boxplots graphed per response category showing the range of vOTU counts (y axis) separated and colored by predicted host taxonomic groups (x axis) across all three plots. The x axis is sorted by rank, but with all Proteobacteria groups adjacent to one another. The box plots represent 75% of the data with the median as a line, and whiskers represent 90% of the data. Box plot colors correspond to microbial taxonomy on phylum level except Archaea.

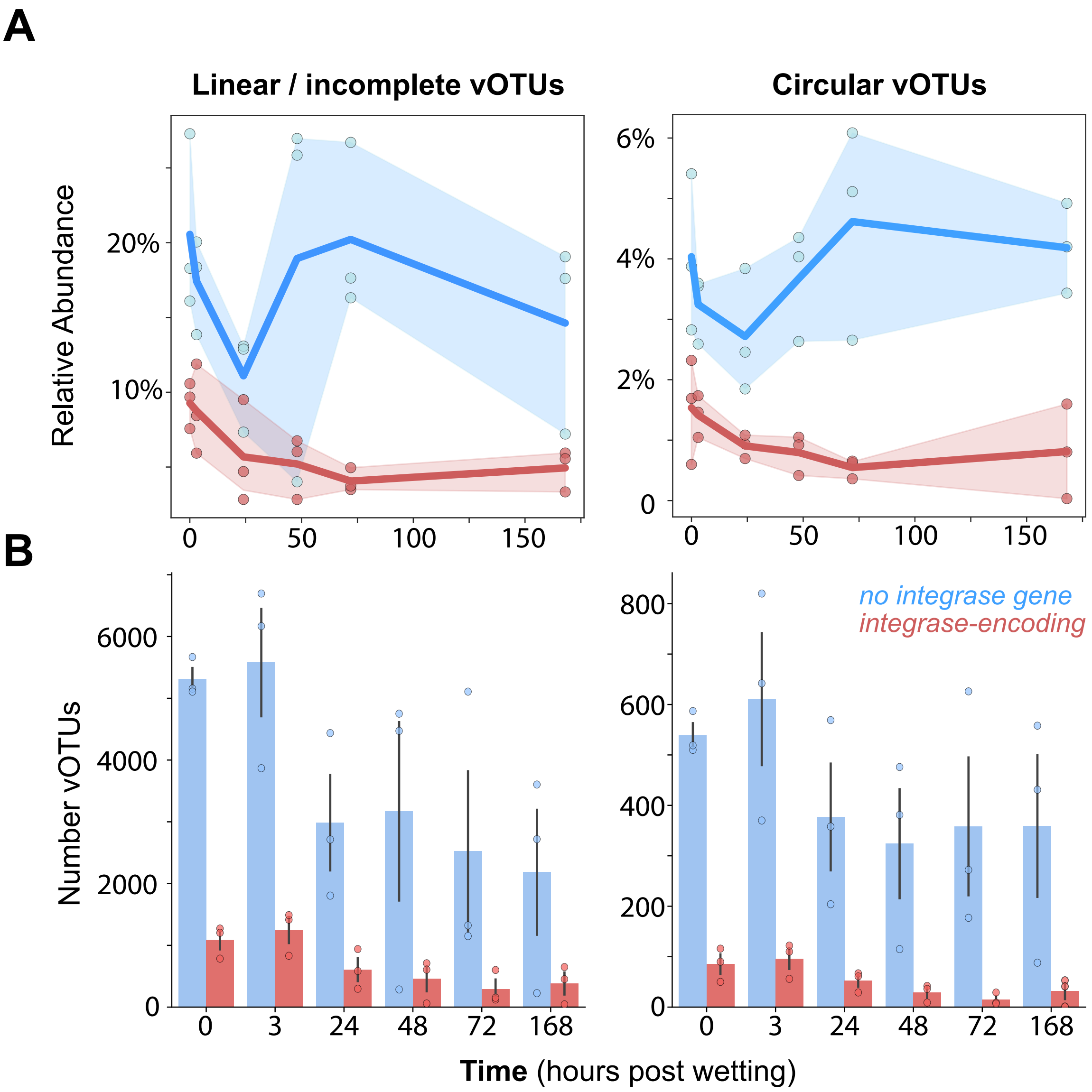

Supplementary Figure 5 | Relative abundance and richness of integrase-encoding vOTUs through time following soil wet-up. vOTUs were split by whether they encode an integrase gene (red) or not (blue) and whether the vOTU is predicted to circularize (right set of graphs) or is linear and perhaps fragmented (left set of graphs). (A) Aggregated relative abundance (percent of total sample reads) of integrase-encoding or not integrase-encoding vOTUs. The error band around each line represents a 95% confidence interval of the mean aggregated relative abundance across triplicate microcosms. Underlying data points show the aggregated relative abundance of vOTUs per microcosm. (B) Counts of integrase-containing and non-integrase containing vOTUs. Error bars represent the standard error of the mean number of vOTUs in each of three replicate microcosms.
