# Supplementary material for: A subset of viruses thrives following microbial resuscitation during rewetting of a seasonally dry California grassland soil
Source: Nat Commun. 2023 Sep 20;14:5835. doi: 10.1038/s41467-023-40835-4 (PMC10511743; doi:10.1038/s41467-023-40835-4)
Supplement: Supplementary file 3 — Description of Additional Supplementary Information [file 41467_2023_40835_MOESM3_ESM.pdf]

<u>Description of Additional Supplementary Files</u>

Supplementary Data 1: Virome DNA extraction yields and sequencing generated per virome and metagenome sample sequenced.
