# Supplementary material for: A subset of viruses thrives following microbial resuscitation during rewetting of a seasonally dry California grassland soil
Source: Nat Commun. 2023 Sep 20;14:5835. doi: 10.1038/s41467-023-40835-4 (PMC10511743; doi:10.1038/s41467-023-40835-4)
Supplement: Supplementary file 5 — Reporting Summary [file 41467_2023_40835_MOESM5_ESM.pdf]

nature portfolio

Corresponding author(s): Steven J. Blazewicz

Last updated by author(s): Aug 1, 2023

Reporting Summary

Nature Portfolio wishes to improve the reproducibility of the work that we publish. This form provides structure for consistency and transparency in reporting. For further information on Nature Portfolio policies, see our Editorial Policies and the Editorial Policy Checklist.

Statistics

For all statistical analyses, confirm that the following items are present in the figure legend, table legend, main text, or Methods section.

n/a	Confirmed	
☐	☒	The exact sample size (*n*) for each experimental group/condition, given as a discrete number and unit of measurement
☐	☒	A statement on whether measurements were taken from distinct samples or whether the same sample was measured repeatedly
☐	☒	The statistical test(s) used AND whether they are one- or two-sided<br>*Only common tests should be described solely by name; describe more complex techniques in the Methods section.*
☒	☐	A description of all covariates tested
☐	☒	A description of any assumptions or corrections, such as tests of normality and adjustment for multiple comparisons
☐	☒	A full description of the statistical parameters including central tendency (e.g. means) or other basic estimates (e.g. regression coefficient) AND variation (e.g. standard deviation) or associated estimates of uncertainty (e.g. confidence intervals)
☐	☒	For null hypothesis testing, the test statistic (e.g. *F*, *t*, *r*) with confidence intervals, effect sizes, degrees of freedom and *P* value noted<br>*Give P values as exact values whenever suitable.*
☒	☐	For Bayesian analysis, information on the choice of priors and Markov chain Monte Carlo settings
☒	☐	For hierarchical and complex designs, identification of the appropriate level for tests and full reporting of outcomes
☐	☒	Estimates of effect sizes (e.g. Cohen's *d*, Pearson's *r*), indicating how they were calculated

Our web collection on statistics for biologists contains articles on many of the points above.

Software and code

Policy information about availability of computer code

Data collection	DNA sequences for viral-enriched metagenomes ("viromes") were collected by performing shotgun sequencing on an Illumina Novaseq (2x150 cycles) (UC Davis). DNA density fractions for stable isotope probing (SIP) were collected via ultracentrifugation (Beckman-Coultier (Indianapolis, IN, USA) VTi 65.2 rotor at 44,100 rpm). Metagenome sequences for SIP and unfractionated sequence assembly were collected on an Illumina Novaseq 2x150 cycles (Novogene). Please see methods section for further details.
Data analysis	All analyses were conducted using Python version 3.8.2 with the scikit package (https://scikit-learn.org) for statistical analyses and the seaborn package (https://seaborn.pydata.org) for data visualization. Additionally, the following software was used: MEGAHIT v1.2.9; BBTools v.39.0; Prodigal v2.6.3; SAMtools v1.17; MaxBin v2.2.7; MetaBAT2 v2.12.1; Concoct v1.1.0; Metawrap v1.3.2; dRep v3.0.1; DAS_Tool v1.1.1; GTDB-tk v1.5.1; CoverM v0.6.1; MinCED v0.4.2; BLAST+ 2.12.0; USEARCH v10.0; VirSorter1 v1.0.6; VirSorter2 v2.2; VIBRANT v1.2.1; deepvirfinder v1.0; seeker v1.0.3; VRCA; DRAM-v v1.2.0; MMseqs2 v13-45111; Kaiju v1.8.0; PropagAtE v1.1.0. Please see methods for further details.

For manuscripts utilizing custom algorithms or software that are central to the research but not yet described in published literature, software must be made available to editors and reviewers. We strongly encourage code deposition in a community repository (e.g. GitHub). See the Nature Portfolio guidelines for submitting code & software for further information.

Data

Policy information about availability of data

All manuscripts must include a data availability statement. This statement should provide the following information, where applicable:

- Accession codes, unique identifiers, or web links for publicly available datasets
- A description of any restrictions on data availability
- For clinical datasets or third party data, please ensure that the statement adheres to our policy

Raw data used in this publication is available on the NCBI short read archive under project submission number PRJNA856348 [https://www.ncbi.nlm.nih.gov/bioproject/?term=PRJNA856348]. Viral genomes can be found on ggKbase https://ggkbase.berkeley.edu/hopland_4th_wedge_virus_set. All qSIP and relative abundance data can be found on the project repository on GitHub [https://github.com/amnicolas/soilviralwet-up]. The Viral-Host Database [https://www.genome.jp/virushostdb/] and a spacer database from Shmakov et al., 2017 [10.1128/mBio.01397-17] were used for additional host-virus matches.

Human research participants

Policy information about studies involving human research participants and Sex and Gender in Research.

Reporting on sex and gender	N/A
Population characteristics	N/A
Recruitment	N/A
Ethics oversight	N/A

Note that full information on the approval of the study protocol must also be provided in the manuscript.

Field-specific reporting

Please select the one below that is the best fit for your research. If you are not sure, read the appropriate sections before making your selection.

☐ Life sciences ☐ Behavioural & social sciences ☒ Ecological, evolutionary & environmental sciences

For a reference copy of the document with all sections, see nature.com/documents/nr-reporting-summary-flat.pdf

Ecological, evolutionary & environmental sciences study design

All studies must disclose on these points even when the disclosure is negative.

Study description	We conducted a rewetting of seasonally dry California grassland soil with natural abundance water and water containing the stable isotope of oxygen (18O) (98 atom% 18O) to comprehensively track 26,368 viral and 542 microbial host populations in response to this environmental perturbation after a dry season. We generated 18 viral-enriched metagenomes (viromes) from six time points – dry soil (0 hours) and 3 hours, 24 hours, 48 hours, 72 hours, 168 hours following wet-up – and 234 metagenomes representing five time points (0 hours, 24, 48, 72, 168) and density fractionated and unfractionated DNA. All viromes and metagenomes per time point were sampled in biological triplicate soil microcosms derived from unique field plots from a field site at the Hopland Research and Extension Center in Hopland, California, USA.
Research sample	Topsoil samples (0-15 cm, roughly 0.5 m3) from replicate field plots were collected from the Hopland Research and Extension Center (HREC) in Northern California on August 28th, 2018 after experiencing mean annual precipitation during the rainy season. These samples were choses because they are well documented to demonstrate a large 'Birch effect' following the first seasonal rewetting event and they represent Mediterranean grasslands. Soil was collected before the first rainfall event of the season and average gravimetric soil moisture was 3%. We sampled this grassland soil following the prolonged summer dry season in order to simulate the first rainfall in the lab.
Sampling strategy	All experiments and analyses were conducted with our three biological replicates per time point to ensure we had the statistical power to run our quantitative stable isotope probing (qSIP) pipeline. Soil samples were collected from 0-15 cm depth (roughly 0.5m3) using an ethanol sterilized trowel and transfered to ziplock bags for transfer to LLNL. Sample size was chosen to have an adequate amount of material for SIP laboratory incubations and to support the downstream analyses (SIP metagenomics, viromics) as our group has previously applied and published.
Data collection	Soil samples were collected by a team of researchers from the Firestone Lab at the University of California, Berkeley and by the Pett-Ridge group at Lawrence Livermore National Laboratory (LLNL). Following field sampling, dry soil was transferred to LLNL where it was homogenized and sieved (2mm) by a team of researchers from the Firestone and Pett-Ridge groups to remove large rocks and roots. Dr. Steven J. Blazewicz and Dr. Alexa M. Nicolas rewetted dry soil microcosms. Xiao Bin Max Li and Marissa Lafler processed soil microcosms for virome and metagenomic sequencing and the LLNL high-throughput stable isotope probing pipeline. Field sampling information was recorded with pen and paper. Incubation details and sample collection were recorded in Microsoft Word and Excel.

Timing and spatial scale	Field plots were established in 2017 and measured 1.8 m x 1.8 m. Circular subplots were surrounded by a 15 cm deep PVC "collar" designed to be fitted with an above-ground cylindrical chamber. Each circular subplot was subdivided into four 15 cm deep sections via plexiglass dividers; this "wedge" design allowed us to destructively harvest soil from a single circular subplot. Soil was removed from 3 replicate subplots, located approximately 3 m apart, on August 28th, 2018. The laboratory wet-up simulation in microcosms occurred following soil processing on September 18, 2018. Samples were destructively harvested at 0 hours (dry soil), 3 hours, 24, 48, 72, 168 hours in coordination with the observed "Birch Effect" (efflux of mineralized carbon from soil). These sampling times were chosen because previous data showed that bacterial growth is measurable within 3 h following rewetting, and the majority of C mineralization stimulation is completed within 168 h.
Data exclusions	No data were excluded from this study.
Reproducibility	All analyses can be reproduced based on the datasets provided. All data analyzed are from biological triplicate samples.
Randomization	Field soil was collected from 3 of 8 replicate field plots, chosen at random. Each field replicate was homogenized and sieved and split into 17 microcosm. 2 microcosms from each field replicate were sampled at random at 0 h for viromes and SIP controls. The remaining microcosms were randomly selected to wet up with natural abundance water as a control (10 cosms) or 18O-enriched water (5 cosms), that were treated the same following wetup, incubated in parallel, and were randomly selected for destructive harvesting at appropriate times (3, 24, 48, 72, 168 h following rewetting. DNA extraction and SIP processing was done in random batches.
Blinding	All resulting viral and microbial sequences from this study were provided unique IDs.

Did the study involve field work? ☒ Yes ☐ No

Field work, collection and transport

Field conditions	Soil was collected on August 28, 2018 from a soil defined as a loamy-skeletal, mixed, superactive, thermix Typic Haploxeralf 55% Squawrock gravelly loan and 30% Witherell loam with 15-50% slopes. During the growing season these field plots were covered by mixed grassland flora with Avena sp. (wild oat) as the most abundant plant. At the time of collection the temperature reached a high of 24.1˚ C, a low of 12.1˚C and had a dew point temperature of 11˚C. There was no precipitation.
Location	GPS 39.004160, -123.086009, Hopland, California, USA<br>Soil cores sampled at 0-15 cm, roughly 0.5 m3, depth
Access & import/export	Hopland Research and Extension Center (HREC) is managed by the University of California Agriculture and Natural Resources (UC ANR). Our research at HREC was allowed after our proposal was thoroughly reviewed and approved by the Research Advisory Committee review board. Professor Mary Firestone maintained a concurrent appointment through the College of Natural Resources with UC ANR which enabled additional benefits and access to HREC. The study site was accessible by paved road and disturbance caused by soil removal was minimized by using small hand tools. Soil from the studied field site is unregulated and was collected and processed within California and did not require a permit for transport.
Disturbance	Small amounts of soil that were removed from the field were backfilled with comparable soil to minimize landscape deterioration.

Reporting for specific materials, systems and methods

We require information from authors about some types of materials, experimental systems and methods used in many studies. Here, indicate whether each material, system or method listed is relevant to your study. If you are not sure if a list item applies to your research, read the appropriate section before selecting a response.

Materials & experimental systems

n/a	Involved in the study
☒	☐ Antibodies
☒	☐ Eukaryotic cell lines
☒	☐ Palaeontology and archaeology
☒	☐ Animals and other organisms
☒	☐ Clinical data
☒	☐ Dual use research of concern

Methods

n/a	Involved in the study
☒	☐ ChIP-seq
☒	☐ Flow cytometry
☒	☐ MRI-based neuroimaging
